# Research Progress of Pyroptosis in Alzheimer’s Disease

**DOI:** 10.3389/fnmol.2022.872471

**Published:** 2022-06-16

**Authors:** Weiyue Xue, Di Cui, Ye Qiu

**Affiliations:** ^1^Department of Physical Education, Hunan University, Changsha, China; ^2^Department of Biology, Hunan University, Changsha, China

**Keywords:** pyroptosis, Alzheimer’s disease, gasdermin, neurofibrillary tangles (NFTs), tau phosphorylation, β-amyloid, NLRP3, caspase

## Abstract

Alzheimer’s disease (AD) is a disease characterized by insidious and progressive neurodegeneration, with clinical syndromes of memory and visuospatial skills damage. The pathogenic mechanism of AD is complex in which neural inflammation and neuron death play important roles. Pyroptosis, an inflammatory programmed cell death, has been reported to be involved in neuron death. Pyroptosis is executed by the protein family of gasdermins which punch pores on plasma membrane when activated by the upstream signals including the activation of NLRP3 and caspases, and subsequently triggers the inflammatory cascades featured by the release of interleukin (IL) -1β and IL-18. Herein, we summarized the current research on the roles of neuron pyroptosis in AD, aiming to provide a comprehensive view of the molecular mechanisms underlying AD pathogenesis and potential therapeutic targets for AD.

## Introduction

As the accelerating of population aging around the world, the diseases of neurodegeneration, such as Alzheimer’s disease (AD), has been posing great threat to the public health and elderly care. According to the global survey in 2020, there were 47 million AD patients worldwide, and until 2030, that number would be predicted reaching to 82 million with approximate 16 million in China (Jia et al., 2011; Meng et al., 2021). AD is the common type of dementia characteristic with extracellular beta-amyloid (Aβ) clustering into amyloid plaques and intracellular tau proteins exaggerating into toxic neurofibrillary tangles (NFTs), while the decreasing synaptic acetylcholine level and neuron dystrophy are also featured in AD symptoms. It has been found that the hippocampus was anatomically the earliest changed encephalic area in AD patients (Duan et al., 2021; Peng, 2021). Scientists do not yet fully understand what causes AD and there is no specific drug to treat this chronic neurodegeneration disease but only ameliorate its symptoms so far. From pathological aspect, aberrant activation of cell death is common featured in AD nosogenesis, facilitating loss of neurons, and cellular dysfunction, while the recently clarified pyroptosis, as an inflammatory programmed cell death, was reported playing a critical role in neuron death (Moujalled et al., 2021). Gasdermin family (GSDMs) serves as the core executors in pyroptosis which punch pores on cell membranes in response to the upstreamed NLRP3 (Nod-like receptor Pyrin domain 3), and caspases (cysteinyl aspartate-specific proteases) signals, triggering inflammatory cascades to release IL-1β, and IL-18 (Shi et al., 2017). Researches showed that reduced inflammasome could decrease GSDMD expression and pyroptosis in neurons to effect neuroprotection (Tan et al., 2014). on the contrary, overexpression GSDME accelerated neuron pyroptosis and inflammatory cell death (Wang, 2019; Liu, 2020). In this review, we summarized the research progress of pyroptosis in AD, aiming to expound novel molecular mechanisms of AD pathogenesis and explore new and potential therapeutic targets for AD therapeutic.

## Pyroptosis

Pyroptosis is known as one of the programmed cell death, and is considered as an important innate immune response in our body. Usually, the occurrence of pyroptosis activates the immune system to eliminate pathogens in the host, while pyroptosis excessive activation aggravates inflammatory reactions implying multiple diseases pathology (Man et al., 2017). Early in 1980s, a type of cell death inducing massive inflammatory responses has been found in macrophages stimulated by toxins or bacterial infections, but considered as apoptosis. Until 1999, Hersh et al. (1999) found that some kind of specific apoptosis could not be activated under caspase-1 knockout condition. That was the very first study of caspase-1 related to pyroptosis, though its activation mechanism was unclear. In order to emphasize its pro-inflammatory feature and distinguish it from traditional apoptosis, this programmed cell death dependent on caspase-1 was firstly proposed by Cookson in 2001 and named as pyroptosis (D’Souza and Heitman, 2001). In 2017, Shao Feng and his research team revealed that a member of gasdermin family, GSDMD, acted as the direct substrate of caspases, and directly executed pyroptosis by inducing plasma membranes pore-formation after the cleavage of caspases; besides, all members of GSDMs except DFNB59 held the ability to punch holes in the membrane, thus pyroptosis was specifically redefined as programmed cell death mediated by GSDMs (Shi et al., 2017). With the deepening of research, now we know although pyroptosis and apoptosis both belong to program cell death, but they are totally differentiated. Pyroptosis is characterized by cell swelling until cell membrane rupture, releasing pro-inflammatory factors, and inflammatory caspase activation leads to cell membrane pore formation, permeability, cell swelling, capsule rupture, and finally release of cell contents, accompanied by IL -1β and IL-18, which activates a much stronger inflammatory response (Jorgensen and Miao, 2015). Unlike pyroptosis, apoptosis is characterized by the shrinkage and concentration of cytoplasm without cell rupture and surrounding inflammatory burdens (Hei and Liu, 2018). Although, for a long time, pyroptosis has been considered as monocyte death mediated by caspase-1 (Bergsbaken et al., 2009). However, the discovery of pyroptosis executor, GSDMs, delivering caspases’ signals to cell death, arouse academic attention on its role in basic life science (Shi et al., 2017). The key molecules involving in pyroptosis and their cascades were described here below.

### Gasdermin Family and Cleavage

There are 6 known members in GSDMs, GSDMA, GSDMB (not in mice), GSDMC, GSDMD, GSDME (DFNA5), and DFNB59, structurally containing both N-terminal and C-terminal except DFNB59 only having C-terminal (Table 1; Shi et al., 2017). The finding of GSDMD serving as the pyroptosis executioner dated back to 2015 by Shao Feng and its activation was followed by upstream molecular, caspase-1 (Shi et al., 2015). GSDMD contains about 480 amino acids, and the gasdermin-N terminal connects gasdermin-C by a long ring region (Wang, 2019). Specifically, caspase specifically recognizes GSDMs, and GSDMD could be cleaved by caspase-1/4/5/11, separating N -terminal pore-forming domain (PFD) from C-terminal repressor domain (RD), and converging PFD to the cell membrane to further activate pyroptosis (Ding et al., 2016; Dong and Shao, 2019). Traditionally, caspase activation has been defined as firstly recognizing the substrates’ tetrapeptide, XXXD (D is aspartic acid, X is other amino acid), and then cutting the specific peptide bond after D. The research of Feng Shao found that the recognition of GSDMD by caspase did not depend on the sequence characteristics of the tetrapeptide, but specifically directly recognized the C-terminal domain of GSDMD (Wang K. et al., 2020). Studies have found that the expression level of GSDMD in the cerebrospinal fluid of AD patients was significantly higher than that of the general control group, and positively related to the release level of inflammatory factors, implying GSDMD could basically be applied as a diagnostic index of AD (Shen et al., 2021). Other than GSDMD, recently, researches confirmed that GSDME also activated pyroptosis. GSDME is located on human chromosome 7 and mouse chromosome 6, with a molecular weight of 54 kDa. GSDME is mainly expressed in the cytoplasm, with a small amount of expression in the nucleus, and was initially found as the deafness gene. In pyroptosis, GSDME was reported been cleaved and activated by upstream caspase-3, and granzyme B (GzmB) was also found to activate GSDME at the same cleavage site as caspase-3 (Zhang et al., 2020). Current research on GSDME mainly focused on cancer treatment and confirmed its tumor suppressive effects, while un-cleavable or pore-defect GSDME proteins could not repress tumor due to inactivating pyroptosis. Besides, GSDME-mediated pyroptosis cause inflammatory cytokine storm, and its role under different physiological and pathological conditions still needs to be further studied and defined (Wan et al., 2022). Researchers in field of neuroscience started to concern GSDMs and pyroptosis in AD pathology, and several preliminary studies affirmed that pyroptosis significantly related to AD, and GSDMD and GSDME were the main targets, which would be specificity focused and discussed hereunder.

**TABLE 1:** GSDM family (Shi et al., 2017; Feng et al., 2018).

GSDMs	Chromosomal location in human	Expression	Activation
GSDMA	17q21.1	Esophagus, stomach, skin	Unknown
GSDMB	17q12	Esophagus, stomach, liver, colon, intestine, T cells	Caspase-3/6/7
GSDMC	8q24.21	Esophagus, stomach, trachea, spleen, skin, intestine	Caspase-3
GSDMD	8q24.23	Esophagus, stomach, intestine, lymphocyte	Caspase-1/4/5/11
GSDME (DFNA5)	7p15	Placenta, cochlea, brain, small intestine	Caspase-3 GzmB
DFNB95	2q31.2	Inner ear, auditory pathway	Unknown

### Caspases

Caspases contains a family of cysteine aspartate-specific proteases members. Caspase-1/4/5/11 has been found involved in pyroptosis: caspase-1 was activated by inflammasome after the cleavage of its precursor (pro-caspase-1); caspase-4/5/11 was directly activated by lipopolysaccharide (LPS); caspase-8 was involved in NLRP3-dependent pyroptosis; caspase-3 activated pyroptosis by targeting GSDME (Saresella et al., 2016; Zhang Y. G. et al., 2021). Pro-caspase-1 consists of three specific structures: CARD domain (caspase recruitment domain, CARD), large subunit P20, and small subunit P10. CARD domain interacts with other CARD proteins, while P20/P10 is key to caspase-1 activation by forming firstly individual heterodimer to combine a tetramer (Wu et al., 2011). Mutation experiments found that conservative self-shearing at the N-terminal of the P10 subunit was critical for GSDMD cleavage (Wang K. et al., 2020). Besides, caspase-3 is not only an apoptosis executor protein, but also existing studies have found that dcaspase-3 could also activate pyroptosis in the present of GSDME (Zhang Y. G. et al., 2021). No matter in apoptosis or pyroptosis, the caspase activation has been verified connecting to cognitive disorder in AD, which will be talked later.

### Pyroptosis Mechanism

**FIGURE 1:**
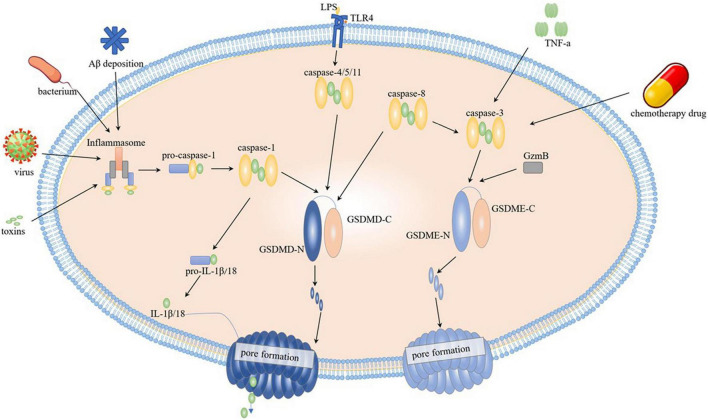
Pyroptosis mechanism. Classical pathway is activated by caspase-1, it is activated by inflammasome, toxins, virus and bacterium through inflammasome; non-classical pathway is activated through caspase-4/5/11 by direct binding to LPS. After caspases’ cleavage on GSDMD or GSDME, the N-terminals translocate on cell membrane resulting in pore formation. Chemotherapy drug and TNF-α acts on caspase-3, and GSDME is activated directly by caspase-3 and GzmB or indirectly by caspase-8.

The activation of pyroptosis triggered by GSDMD is divided into classical and non-classical pathways according to different upstream signals (Figure 1). The classic pathway activates caspaes-1 *via* inflammasome. Concretely, pattern recognition receptors (PRRs) bind to related pattern recognition molecules to activate inflammasome, and pathogen-associated molecular patterns (PAMPs), and damage-associated molecular patterns (DAMPs) can be separately activated by PRRs (McIntire et al., 2009). After inflammasome activation, they cleave pro-caspaes-1 into caspaes-1, and then caspase-1 cleaves GSDMD into two parts, C-terminal and N-terminal, and in quick succession GSDMD-N terminals make holes on cell membrane triggering pyroptosis. Interestingly, the cleaved GSDMD-N terminals activates NLRP3 inflammasome in reverse to cleaved pro-IL-1β and pro-IL-18 for further releasing IL-1β and IL-18 (Feng et al., 2018). NLRP3 is one of the most hackneyed core molecules of inflammasome (Hei and Liu, 2018). In addition to that, NLRP1, NLR family CARD domain containing 4 (NLRC4), absent in melanoma 2 (AIM2), and Pyrin proteins have been reported appearing successively in caspase-1-dependent inflammasome (Ji et al., 2020). In AD brain, Aβ deposition is one of the main factors of inflammasome formation. The non-classical pathway directly activates caspase-4/5 in human and caspase-11 in mice on the occasion of lipopolysaccharide (LPS), and activated caspases move forward the GSDMD step facilitating the cell membrane pore formation and triggering pyroptosis, during which process Toll-like receptor 4 (TLR4) located on cell surface serving as the receptor of LPS (Ji et al., 2020). It has been found that caspase-8 forthright sheared GSDMD at D276 location, and other relevant mechanisms need to be further elaborated (Orning et al., 2018). Respectively, the pyroptosis mediated by GSDME is primarily activated by both caspase-3 and GzmB. When cells were stressed under stimulation of chemotherapeutic drugs or inflammatory factor tumor necrosis factor (TNF) -α, caspase-3 activated and further cut and activated GSDME (Wang et al., 2017). What cannot be ignored is, caspase-3 activation could also be achieved by upstream caspase-8 to mediate IL-1β release (Zeng et al., 2019). GzmB specifically cut GSDME, and its cleavage site is consistent with caspase-3 and, similarly with GSDMD, cleaved GSDME-N terminals gather on the cell membrane to make holes and finally lead to pyroptosis (Zhang et al., 2020).

## Pyroptosis in Alzheimer’s Disease

Pyroptosis is a newly discovered programmed cell death and has been found involved in the pathological mechanism of AD (Shen et al., 2021). Inflammasome and pro-inflammatory cytokines in the activation pathway of pyroptosis have been confirmed related to the pathogenesis of AD, meanwhile the AD hallmarks, Aβ and tau proteins, were proven involving in the occurrence of pyroptosis with unclear mechanism (Saresella et al., 2016; Shen et al., 2021). Therefore, it is of great theoretical significance to study the role of pyroptosis in AD, and the present article summarized the recent research progress of pyroptosis in AD aiming to expound novel molecular mechanisms of AD pathogenesis and explore new and potential therapeutic targets for AD therapeutic (Table 2).

**TABLE 2:** Pyroptosis in AD.

Works	Key molecules	Summary
Tan et al. (2014)	Aβ, NLRP1, IL-1β	Aβ deposition increased NLRP1. Inhibiting NLRP1 decreased caspase-1 and IL-1β.
Wu et al. (2013)	Aβ, NLRP3, IL-1β	In Microglial, IL-1β was activated through Aβ/caspase-1/NLRP3 pathway.
Yap et al. (2019)	Aβ	Aβ deposition activated inflammasome, and then cleaved caspase-1 triggering pyroptosis.
Venegas et al. (2017)	Aβ	ASC bound to Aβ, resulting in the formation of Aβ oligomers and aggregates.
Li J. et al. (2020)	Aβ, GSDMD	Inhibiting NLRP3 reduced pyroptosis and decreased NLRP3, caspase-1, GSDMD.
Han et al. (2020)	Aβ, GSDMD	Aβ1-42 induced pyroptosis through NlRP3/caspase-1/GSDMD signaling pathway.
Heneka et al. (2013)	Aβ, GSDMD	Aβ deposition induced NLRP3 inflammasome. NLRP3 reduction decreased caspase-1 and GSDMD.
Li et al. (2021)	GSDMD	Guanidine reduced hippocampal neuron damage by inhibiting GSDMD mediated pyroptosis.
Lei et al. (2021)	Caspase-8, GSDME	Hippocampal injured through caspase-8/GSDME pyroptosis pathway.
Liu (2020)	GSDME	Hippocampal CA3 region was found neuronal degeneration and cell death with increased GSDME positive cells.
Feng et al. (2021)	Tau protein, IL-1β	IL-1β induced microglial phagocytic activity loss, and tau protein hyperphosphorylation.
Ising et al. (2019)	Tau protein, NLRP3	NLRP3 induced tau protein hyperphosphorylation and aggregation.
Li Y. et al. (2020)	Tau protein, caspase-1	Injecting caspase-1 inhibitor or lithium chloride suppressed tau protein hyperphosphorylation and pyroptosis.
Saresella et al. (2016)	NLRP1, IL-1β, IL-18	NLRP1/caspase-1/ILs pathway activated in AD brain.
Ojala et al. (2009)	Inflammatory cytokines	IL-1, IL-1β and IL-18 expression was increased in AD brain.

### Inflammasome and Pro-inflammatory Factors in Alzheimer’s Disease

NLRP1 and NLRP3 were found up regulated in AD patients, implying the classical pyroptosis pathway in AD pathology. NLRP1, the first member of discovered nod-like receptor (NLR) family, participates into constitution of NLRP1 inflammasome, together with connector protein ASC, and pro-caspase-1, and NLRP1 is highly conservatively expressed in brain vertebral neurons and oligodendrocytes (Tan et al., 2014). NLRP1 activates caspase-1 and next drives inflammatory response and pyroptosis (Masters et al., 2012). Studies showed NLRP1 expression was up-regulated in the brain tissue of APP/PS1 mice, however, using NLRP1 inhibitor decreased caspase-1 and IL-1β expression, reduced neuron pyroptosis, and finally improved cognitive impairment (Tan et al., 2014). Furthermore, studies on relationship between NLRP1 and AD found that the expression of NLRP1 in AD mice was significantly up-regulated, and NLRP1 induced downstream inflammatory cascades through activation of caspase-1, maturation of IL-1β and IL-18 and neuronal death, which further led to the occurrence of AD (Saresella et al., 2016). Based on these effects, we hypothesized that NLRP1 involved in the neuron inflammatory pyroptosis during AD process, and blocking pyroptosis through NLRP1 positively influence AD pathogenesis. NLRP3, another important member of the NLR family, became a novel research hotspot in the field of pyroptosis of late years. Similarly to NLRP1 inflammasome, NLRP3 inflammasome consists of connector protein ASC, pro-caspase-1, and NLRP3, and NLRP3 is reported mainly expressed in microglia (Hei and Liu, 2018). During AD progression, NLRP3 inflammasome activated caspase-1 to mediate GSDMD cleavage in microglia, facilitating IL-1β and IL-18 releasing (Feng et al., 2021). Inhibiting NLRP3 by MCC950 significantly reduced the deposition of amyloid beta in brain and the neurotoxicity of Aβ1-42 because of pyroptosis blocking (Li J. et al., 2020). Briefly, inhibiting of inflammasome cuts down pyroptosis and further alleviates AD pathology. Besides the inflammasome of pyroptosis initiation, its terminal pro-inflammatory factors or cytokines released through cell pores are also critical for AD pathology, such as IL-1β and IL-18 (Man et al., 2017). They were cleaved by caspase-1 from pro-IL-1β and pro-IL-18, and ultimately released extracellularly (Hei and Liu, 2018). Further study found that caspase-1 cleaved pro-IL-1β and pro- IL-18 on the premise of NLRP1/3 (Freeman and Ting, 2016). IL-1, IL-1β and IL-18 were reported high expressed in AD and related to its pathogenesis (Ojala et al., 2009). High-level of IL-1β in microglial caused phagocytic activity loss and tau protein hyperphosphorylation, reduced synaptic plasticity, and resulted in the neurological impairment on learning and memorizing (Saresella et al., 2016). Still in microglial, IL-1β activation based on Aβ/caspase-1/NLRP3 pathway, and Aβ excessive accumulation aggravated AD procedure (Wu et al., 2013). In a short summary, both the initiated inflammasome and terminal inflammatory cytokines are involved in the pathogenesis of AD, hinting the pivotal role of pyroptosis in dementia.

### Aβ and Pyroptosis

The acknowledged pathological hall marker of AD is senile plaque, which is formed through the deposition of Aβ in the brain. More and more researches confirmed that Aβ induced activation of microglia and mediated inflammatory cytokines release (Blasko et al., 2004). Aβ issued a command of pyroptosis, and involved in its mechanism. Han et al. (2020) showed that Aβ1-42 induced pyroptosis through NLRP3/caspase-1/GSDMD pathway. Furthermore, inflammasome was also the downstream of Aβ, in which process Aβ deposition activated the intracellular inflammasome, mediated cleavage of caspase-1, and then triggered pyroptosis, contributing to AD development (Yap et al., 2019). Research in rodent found that the increased NLRP1 expression was attributed to Aβ deposition, and loss-function treatment by NLRP1 siRNA decreased Aβ-dependent pyroptosis (Tan et al., 2014). The same results are also applied to NLRP3. After Aβ deposition, the NLRP3 inflammasome activated, however, NLRP3 could also conversely promote Aβ clearance and improve spatial memory dysfunction by reducing caspase-1 and GSDMD (Heneka et al., 2013). In addition, it has been reported that ASC can bind to Aβ, resulting in the formation of Aβ oligomers and aggregates, and further expansion of Aβ -induced lesions (Venegas et al., 2017). These studies suggest that Aβ induces pyroptosis mainly through the classic pyroptosis pathway of inflammasome caspse-1, and Aβ deposition can induce activation of inflammasome and trigger pyroptosis in brain. However, the effect of pyroptosis in neurons and other neurocytes, such as microglial, still needs to be further studied.

### Tau Protein and Pyroptosis

The abnormal aggregation of tau protein hyperphosphorylation leading to the appearance of NFTs is another acknowledged key pathological phenomenon of AD. Tau protein is ubiquitously expressed in neurons of the central nervous system and serves as a microtubule-related protein participating in the regulation of the tubulin stability (Wang J. C. et al., 2020). Similar to pyroptosis, NLRP3 inflammasome is the upstream signal of tau protein, and NLRP3 was considered to play a key role in AD pathogenesis of tau protein (Ising et al., 2019). Research have found that the activation of NLRP3 could induce tau hyperphosphorylation and excessive aggregation, and the activation degree of NLRP3 in the brain of Tau22 mouse model was higher than that in wild-type; interestingly, hippocampus injection with brain homogenate containing Aβ could induce tauopathy in Tau22 mice, however, knocking out of ASC or NLRP3 improved amyloid plaque pathology in APP/PS1 transgenic mice (Ising et al., 2019). These data indicated that not only NLRP3 but also Aβ itself was involved in the pathological mechanism of tau protein induced AD. Li Y. et al. (2020) applied drugs (FSK and STZ) to mimic tau protein hyperphosphorylation in mice brain, and then confirmed that inhibiting caspase-1 alleviated cognitive disorder and neuron damage; nevertheless, directly inhibiting tau protein hyperphosphorylation by lithium chloride reduced caspase-1, IL-1β, and IL-18 expression, and blocked pyroptosis. *In vitro* studies showed that using specific blocker to inhibit neuropyroptosis improved tau pathology (Sui et al., 2021). Again, the effect of tau protein on pyroptosis has been proven, but the inherent molecular mechanism needs to be further elaborated.

### Pyroptosis in Hippocampus

In AD patients, the hippocampus is the most vulnerable area of the brain injury. The number of GSDME positive cells was increased, and the hippocampal CA3 region was found neuronal degeneration and cell death after disposing with methamphetamine (METH, a dementia simulant), indicating that GSDME-mediated pyroptosis occurred in hippocampal neurons (Liu, 2020). After injection of AAV9-siRNA-caspase-1, the behavior of AD mice in cognitive function experiments was alleviated, and the expressions of NLRP3, caspase-1, and GSDMD were down regulated in both cerebral cortex and hippocampus (Han et al., 2020). It has been reported that guanidine reduced hippocampal neuron damage by inhibiting GSDMD-mediated pyroptosis (Li et al., 2021). These studies suggested that both GSDMs and pyroptosis -related proteins were expressed in the hippocampus, and inhibition of pyroptosis-related proteins could reduce hippocampus damage and play a positive role in the treatment of AD.

In summary, in the AD brain, Aβ deposition induced pyroptosis through classical inflammasome/caspase-1/GSDMD pathway. After NLRP1 and NLRP3 activation, tau protein hyperphosphorylation occurred and aggregated leading to AD pathological mechanism. In addition, GSDMs facilitated pore formation on the cell membrane, and inflammatory cytokines went through these holes to generate inflammatory response.

## Application of Targeting Pyroptosis in the Treatment of Alzheimer’s Disease

Currently, only cholinesterase inhibitors and N-methyl D-aspartate (NMDA) antagonists have been approved for the treatment of AD (Breijyeh and Karaman, 2020). The choline hypothesis suggests that reduced acetylcholine synthesis leads to the development of AD, so increasing cholinergic levels is considered as one of the therapeutic strategies to improve cognition and nerve cell function. Over-activation of NMDA receptors contributes to abnormal calcium signaling and glutamate excessive stress, resulting in synaptic dysfunction and neuronal cell death. NMDA antagonists have been utilized to treat moderate and severe AD (Companys-Alemany et al., 2020). Theoretically, in terms of the drug therapy targeting pyroptosis for AD, the upstream molecules of GSDMD attracts the attention in the field of traditional Chinese and Western medicine to test on prescriptions, drugs, and molecular inhibitors. Methylene blue was found to reduce the inflammatory factors of IL-1β and IL-18 and inhibit the up-regulation of NLRP3 inflammasome in microglia among AD clinical trials (Yan et al., 2020). Progesterone has been reported inhibiting the activation of Aβ -induced NLRP3/caspase-1 inflammasome, and playing a neuroprotective role. Traditional Chinese medicine has been proven effective in the treatment of AD. Baicalin inhibited neuroinflammatory through suppressing NLRP3 inflammasome activation (Jin et al., 2019). Saffron extract (IIIM-141) could inhibit the activation of NLRP3 inflammasome and promote the clearance of Aβ by increasing p-glycoprotein expression (Bharate et al., 2018). The application of chicory flavonoids could inhibit Aβ-induced scorch decay in the hippocampus (Zhao et al., 2019). Bushenhuoxue acupuncture alleviated the cognitive defect by inhibiting NLRP1 and Aβ deposition in mice (Zhang T. et al., 2021). Salidroside effected on pyroptosis and ameliorating AD through NF-κB/NLRP3/caspase-1 pathway to mitigate Aβ 1-42 and D-gal/AlCl_3_ which was induced during hippocampus neuronal injury. Some other anti-inflammatory and antibacterial Chinese herbal medicines have also been found to treat AD by targeting pyroptosis through inhibiting NLRP3 and Aβ signaling pathways, such as Rhizoma coptidis and Schisandra chinensis (Zhu et al., 2019; Kim et al., 2020). In addition, specific inhibitors were reported to improve AD pathology. As mentioned above, neurotoxicity of Aβ1-42 was reduced with the NLRP3 inhibitor MCC95, and spatial memory and brain morphology were improved accordingly. These studies indicate that drugs targeting GSDMs and pyroptosis potentially inhibit the deposition of Aβ and NLRP3 expression effecting on AD alleviation, while the behind pharmacological mechanisms are still unclear.

## Summary and Prospect

In conclusion, current studies have reported that pyroptosis plays important roles in the progress of AD. Caspase-dependent GSDMD acts as the executive protein of pyroptosis, and inflammasome, Aβ, Tau protein, pro-inflammatory cytokines and other molecules related to the pathogenesis of AD are involved in its upstream implying the targeting on pyroptosis can be the next therapeutic target for alleviating AD pathology.

Currently, researchers have already started to study on the correlation between non-drug treatments and pyroptosis, such as exercise and life behaviors. resistance training and aerobic training can significantly reduce pyroptosis-related molecular in the hippocampus of mice, suggesting exercise effectively alleviates the pyroptosis in hippocampal (Bian et al., 2020; Ji et al., 2020). While the specific mechanism of exercise-mediated pyroptosis to improve AD is still unclear, mainly through improving the pathogenesis of AD by down-regulating NLRP3 and other related inflammatory factors and inhibiting the occurrence of pyroptosis of GSDMD. Existing researches confirmed effects of exercise on AD related to Aβ deposition and tau protein hyperphosphorylation, while there is still no attempts on the study on exercise, pyroptosis and their correlation to Alzheimer’s disease. Secondly, in the studies on the pathological mechanism of pyroptosis mediated by AD, the pyroptosis signaling pathway mediated by GSDMD is relatively clear, while there is few study on GSDME mediated signaling pathways, and caspase-3 mediates GSDME to activate pyroptosis. Although current studies focused on the field of tumor therapy, while GSDME could also trigger cytokine storms by forming inflammatory responses, and a large amount of GSDME expression has been observed in the damaged hippocampus. Whether GSDME-mediated pyroptosis is one of the targeting pathways leading to the occurrence and development of AD needs to be further studied urgently.

## Author Contributions

WX and DC conceived of the presented idea. DC and YQ encouraged WX to investigate and supervised the findings of this work. All authors discussed the article writing and contributed to the final manuscript.

## Conflict of Interest

The authors declare that the research was conducted in the absence of any commercial or financial relationships that could be construed as a potential conflict of interest.

## Publisher’s Note

All claims expressed in this article are solely those of the authors and do not necessarily represent those of their affiliated organizations, or those of the publisher, the editors and the reviewers. Any product that may be evaluated in this article, or claim that may be made by its manufacturer, is not guaranteed or endorsed by the publisher.
